# Impact of tandem autologous stem cell transplantation and response to transplant in the outcome of multiple myeloma

**DOI:** 10.1186/2162-3619-1-35

**Published:** 2012-11-26

**Authors:** Rui Bergantim, Fernanda Trigo, José E Guimarães

**Affiliations:** 1 Service of Clinical Hematology, Hospital São João, Porto, Portugal; 2 Faculty of Medicine, University of Porto, Porto, Portugal

**Keywords:** Multiple myeloma, Bone marrow transplantation, Autologous stem cell transplant, Tandem transplant

## Abstract

**Background:**

Multiple Myeloma (MM) is the commonest indication for autologous stem cell transplantation (ASCT).

**Methods:**

We retrospectively analysed data from 85 patients with MM submitted to ASCT in our centre from 2000 to 2010: 132 ASCT were realized, 80 of them as tandem.

**Results:**

After induction, 17.6% were in complete remission (CR), 41.2% in very good partial remission (VGPR) and 41.2% in partial remission (PR). After transplant 44.7% were in CR, 15.3% in VGPR and 40% in PR. With 22 months (range – 3 to 117 months) of median follow-up, median overall survival (OS) was 43 months and progression-free survival (PFS) 22 months. At 5 years, OS was 45.3% (36.7-53.9%, 95%) and PFS 24.5% (18-31%, 95%). Patients with CR after ASCT had significantly longer PFS as compared to patients with PR (27 *vs* 7 months; p = 0.034) but not when compared to patients with VGPR (27 *vs* 19 months, p = 0.485). The *tandem* approach represented an advantage in OS and PFS when compared to only one ASCT (31 *vs* 19 months - p = 0.018, and 40 *vs* 31 - p = 0.04, respectively).

**Conclusions:**

Our results highlight the impact of response to transplant in patients PFS and tandem modality showed to carry better PFS and OS then the single transplant.

## Introduction

Multiple Myeloma (MM) is a neoplasm characterized by abnormal proliferation of plasma cells and secretion of monoclonal immunoglobulin in blood and/or urine [1,2]. It is responsible for 1% of cancers in general and 10-15% of hematologic, accounting for 20% of all deaths due to hematologic malignancies. It affects 4.3/100000 persons per year worldwide and the incidence increases with age (median age 65 years). Around 90% of cases occur after the age of 50. It is more common in male than females (1.4:1) and in Afro-Americans compared to Caucasians (2:1) [1,3].

Signs and symptoms vary greatly and clinical presentation may range from cases that are detected on routine screenings to severe hematologic emergencies [1]. In addition to the classic prognostic classification systems *DurieSalmon Staging* (DSS) [4] and *International Staging System* (ISS) [5]^,^ currently the stratification risk according to cytogenetic group assumed a central role in the prognosis and therapeutic decision [6].

MM is a virtually incurable disease but over the past 30 years we have seen a number of developments in the therapeutic approach of patients with MM that tend to transform it from a rapidly fatal into a more chronic disease [1,2,7,8].

The first milestone of this development, around 1980, was the introduction of high-dose chemotherapy with autologous stem cell rescue as consolidation after induction chemotherapy (QT), improving the progression-free survival and overall survival of MM patients when compared to regimens exclusively using non-myeloablative chemotherapy [1-3,9-13]. The role of ASCT is nowadays taken as the *state-of-the-art* therapeutic which should be offered to patients under 70 years with no important comorbidities. Strategies such as tandem auto-auto and the auto-allogeneic were developed to improve the results of achieved with ASCT. MM is currently the main indication for ASCT in Europe and in the United States [14,15].

Melphalan is the drug of choice for myeloablation in ASCT, based on the knowledge that alkylating agents cause immunosuppression and allow restoring functionality of bone marrow after infusion of hematopoietic progenitors [2].

Despite the improvement in treatment methodology over the 20 years that followed the introduction of ASCT, we witnessed on the early 2000’s another breakthrough when the first results on the use of the entitled *new drugs* - thalidomide [16-19] bortezomib and lenalidomide [20,21] – in induction therapy for MM were published, showing a large improvement in the rate of complete responses without major toxicity. These new drugs are associated with superior disease-free survival, but still no proven benefit on overall survival was demonstrated [22-24]. We are watching a paradigm shift on the natural history of MM, supported by a sequential therapy that allows facing MM as a cancer that is evolving to a chronic disease.

Parallel to this development, our department shaped its therapeutic attitude to offer the most effective solution for its patients. From year 2000 onwards, MM became the main indication to ASCT in our department, with a median of 25 ASCT/year and increasing. In this study, we describe our experience in hematopoietic transplantation in MM over the period 2000 to 2010.

## Design and methods

Patients aged less than or equal to 70 years with a suitable ECOG *performance status*[25] and without significant comorbidities or multiple organ dysfunctions were eligible for ASCT, after induction QT, once they had reached at least a partial response.

Peripheral blood progenitor cells (PBPC) were collected after priming with high-dose cyclophosphamide (4 g/m^2^) and mobilization with granulocyte growth factor (G-CSF) 10 g/kg/day until the last day of apheresis [26,27]. This was performed when the number of CD34+ cells presented to be higher or equal to 10 cells/l and with a minimum target of 2.0x10^6 CD34+ cells/kg, and, if possible, enough to ensure at least two autologous stem cell transplants.

After PBPC collection, patients underwent ASCT conditioning with melphalan 100 mg/m^2^/day for two consecutive days, with reduced dose (70 mg/m^2^/day) for renal insufficiency (creatinine > 2.0 mg/dL) or patients older than 65 years [28]. Infusion of PBPC occurred 24 hours after the end of melphalan. Fluconazole 400 mg/day and Acyclovir 800 mg/2 times per day were given as anti-infectious prophylaxis. No antibacterial prophylaxis was used. Support transfusion of concentrated red blood cells and/or platelets were provided as needed, to a threshold of hematocrit of 26% and platelets of 20x10^9/L, respectively. Until 2006, G-CSF 5ug/kg/day was administered until hematologic engraftment, defined as neutrophil recovery above 0.5x10^9/L in three consecutive days and platelets greater than 20x10^9/L in seven consecutive days without transfusion support. No patient underwent maintenance therapy.

The evaluation of response to ASCT was made around day 100 (+/−10), according to the criteria of the *International Myeloma Working Group* (IMWG) [29]. From 2000 to 2004, patients were only submitted routinely to one transplant. From 2005 to 2009, patients were enrolled in the tandem modality and a second transplant was planned for 3–6 months after the first graft, following the same conditioning regimen and supportive care. For the last 2 years of the period, patients were assigned to tandem transplant only if they had not achieved at least a VGPR.

Regimen related toxicities were classified according to the *Common Terminology Criteria for Adverse Events* - *National Cancer Institute* (NCI-CTCAE) [30]. Transplant related mortality (TRM) refers to any death in the first 100 (+/−10) days after ASCT, whose cause has been directly attributed to the disease or complication over transplantation.

Progression-free survival (PFS) was calculated from the date of transplant to the date of progression/relapse or death, and overall survival (OS) was calculated from the date of transplant to the date of death from any cause.

We conducted an observational retrospective analysis of 132 transplants performed consecutively from 2000 to 2010, inclusive. The endpoints analysed included response after ASCT, PFS, OS, TRM and regimen related toxicities. Another objective of this study included the comparison of *tandem vs* single transplant modality and the impact of the use of G-CSF after the stem cell infusion.

Demographics and baseline characteristics as well as statistical analysis were performed in the *Statistical Package for the Social Sciences* (SPSS) v.18. The *Kaplan*-*Meier method* was used to estimate PFS and OS, and time curves compared by *log*-*rank* test with a confidence interval of 95%. Our study was approved by the institutional review board of our center and it was designed according the tenets of the Declaration of Helsinki. Written informed consent was obtained from the patient for publication of this report and any accompanying images.

## Results

### Patients characteristics

From 2000 to 2010, 85 patients were transplanted (50.6%) male. The median age at transplant was 56 years (range 37–69 years). Table 1 summarizes patient’s clinical characteristics prior to the first ASCT.

**Table 1:** Clinical characteristics of the 85 patients prior to the first ASCT

		**85 patients, n (%)**
Type MM	IgG kappa	34 (40.0%)
	IgG Lambda	14 (16.5%)
	IgA kappa	10 (11.8%)
	IgA Lambda	8 (9.4%)
	Gammopathy with two monoclonal components	4 (4.7%)
	Light Chain MM	11 (12.9%)
	Non Secretor MM	4 (4.7%)
ISS score	I	15 (17.6%)
	II	47 (55.3%)
	III	20 (23.5%)
	Unknown	3 (3.5%)
Durie-Salmon stage	IA	7 (8.2%)
	IIA	16 (8.8%)
	IIIA	49 (57.6%)
	IIB	3 (3.5%)
	IIIB	10 (11.8%)

*Abbreviations*: *MM* – *Multiple Myeloma*; *Ig* – *Immunoglobulin*; *ISS* – *International Staging System*.

## Previous treatments and induction chemotherapy

MM patients had induction therapy either in our centre or were referred for transplant from other hospitals, but in any case they were evaluated prior to transplant. At the time of first ASCT all patients had achieved at least a partial response. There were no patients with refractory or progressive disease at the moment of transplantation.

Prior to ASCT, patients had received 1 to 3 lines of therapy, with 64.7% (n = 55) of them receiving only one line. For induction chemotherapy, 32 (37.6%) patients were treated with idarubicin and dexamethasone, 18 (21.2%) with bortezomib and dexamethasone, 14 (16.5%) with thalidomide and dexamethasone, 16 (18.8%) with vincristine, adriamycin and dexamethasone, 5 (5.9%) with vincristine, idarubicin and dexamethasone. Of all patients, 8.2% underwent previous radiation therapy due to plasmacytomas with cord compression.

## Peripheral blood progenitors cell mobilization, transplant and kinetics of engraftment

All patients underwent peripheral blood progenitors cells mobilization with high-dose cyclophosphamide and G-CSF. After 1–4 apheresis (median of 2), a sufficient number of CD34 + cells was reached to support at least two ASCT (>2x10^6 CD34+ cells/kg x 2). The median number of total CD34 + cells infused was 8.24x10^6 CD34+ cells/kg (range, 3.17 to 5.27x10^6 cells/kg), either for first or second transplant.

Forty patients (47.1%) underwent autologous transplant in a *tandem* modality, 38 patients (44.7%) had only one ASCT and 7 patients (8.2%) were retransplanted after documented relapse and following reinduction. Median time between each ASCT in patients undergoing *tandem* modality was 4 months (range 3–6 months).

For first ASCT, median time to neutrophil engraftment was 16 days (range, 8-49 days) and to platelets was 13 days (range, 5–41 days). In patients undergoing a second ASCT, there was an equally effective engraftment with a median of 16 days (range, 3–24 days) for neutrophils and 13 days (range, 2–22 days) for platelets.

Forty-four patients (51.8%) received G-CSF in the post-transplant period until documented engraftment. There was no statistically significant association between the use of G-CSF and either the number of days of hospitalization or the kinetics of platelet and neutrophil engraftment. Also in respect to infectious complications and relapse no association was found with G-CSF utilization.

## Regimen related toxicity and mortality

Severe toxicity (greater than grade II) occurred in 72 patients (84.2%). The most frequent toxicity was oropharyngeal mucositis (67%, n = 57) and febrile neutropenia/infectious complications (78.8%, n = 67). Two patients required invasive ventilation due to septic shock and were admitted to the intensive care unit with full recovery to the baseline status. There were no deaths related to the ASCT.

## Clinical response

After induction therapy, 15 patients (17.6%) achieved Complete Response (CR), 35 patients (41.2%) Very Good Partial Response (VGPR) and 35 patients (41.2%) Partial Response (PR). On the day 100 following the first ASCT we observed an improvement in CR rate of patients who were previously in VGPR or PR. Of patients transplanted in CR, 13 (86.7%) remained in CR and two (13.3%) lost the CR to VGPR; of those who were not in CR at the time of ASCT, CR was achieved in 25 patients (35.8%), VGPR was achieved in 33 patients (47.1%) and PR was maintained in 12 patients (17.2%). Overall, after ASCT, CR rate was 44.7% (n = 37), VGPR rate 41.2% (n = 45) and PR rate 15.3% (n = 13).

**Figure 1:**
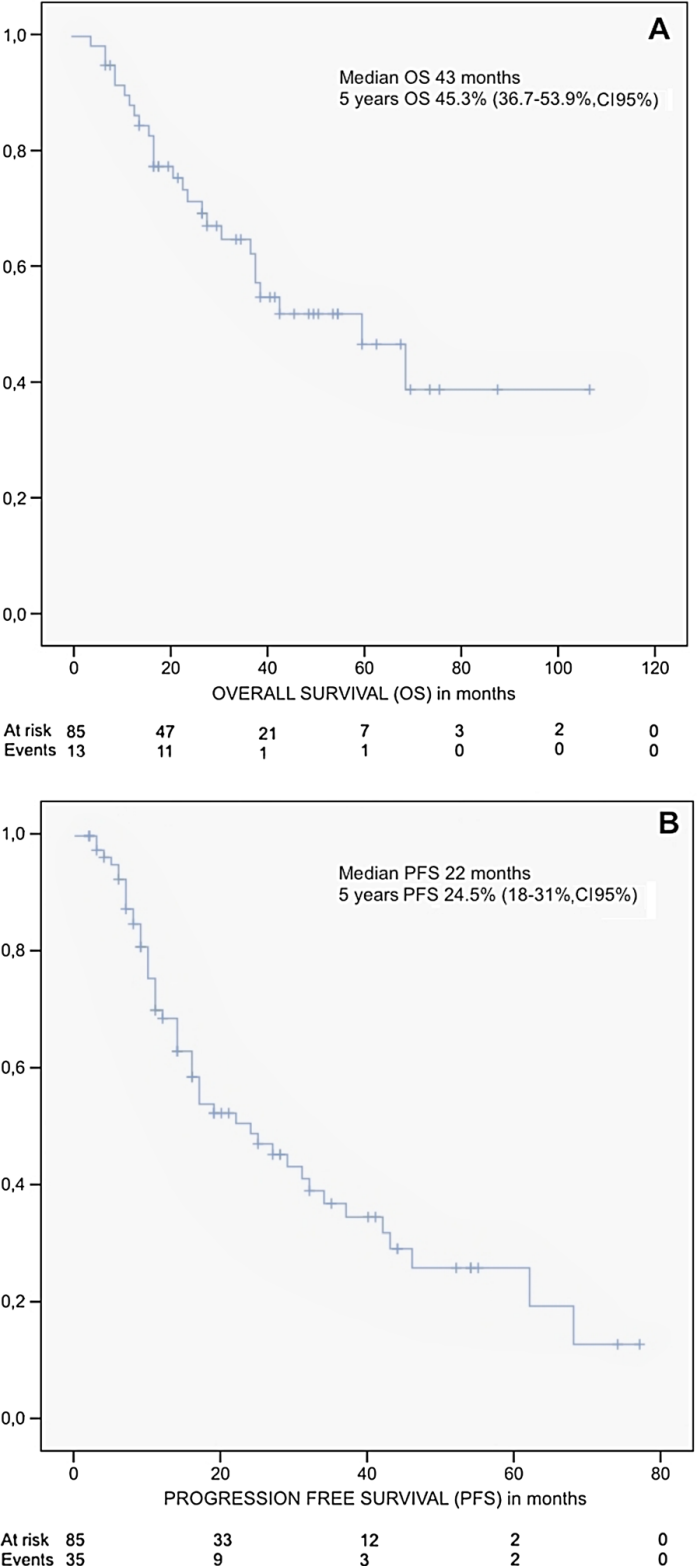
Kaplan Meyer curves for Overall survival (A) and Progression free survival (B) from patients with Multiple Myeloma who underwent autologous stem cell transplantation between 2000 and 2010 in our centre.

With a median follow-up of 22 months (range 3 to 117 months) since the first transplant, for all the 85 patients included in our study median OS was 43 months and PFS 22 months. At 5 years, OS was 45.3% (36.7-53.9%, 95% c.i.) and PFS was 24.5% (18-31%, 95% c.i.) (Figure 1).

New drugs (thalidomide and bortezomib) in induction showed no improvement on achieving a CR before transplant (CR 16.6% *vs* 18.8%) and no difference was observed in terms of OS (median of 43 months *vs* 43 months, p = 0.41) or PFS (median of 31 months *vs* 17 months p = 0.43).

**Figure 2:**
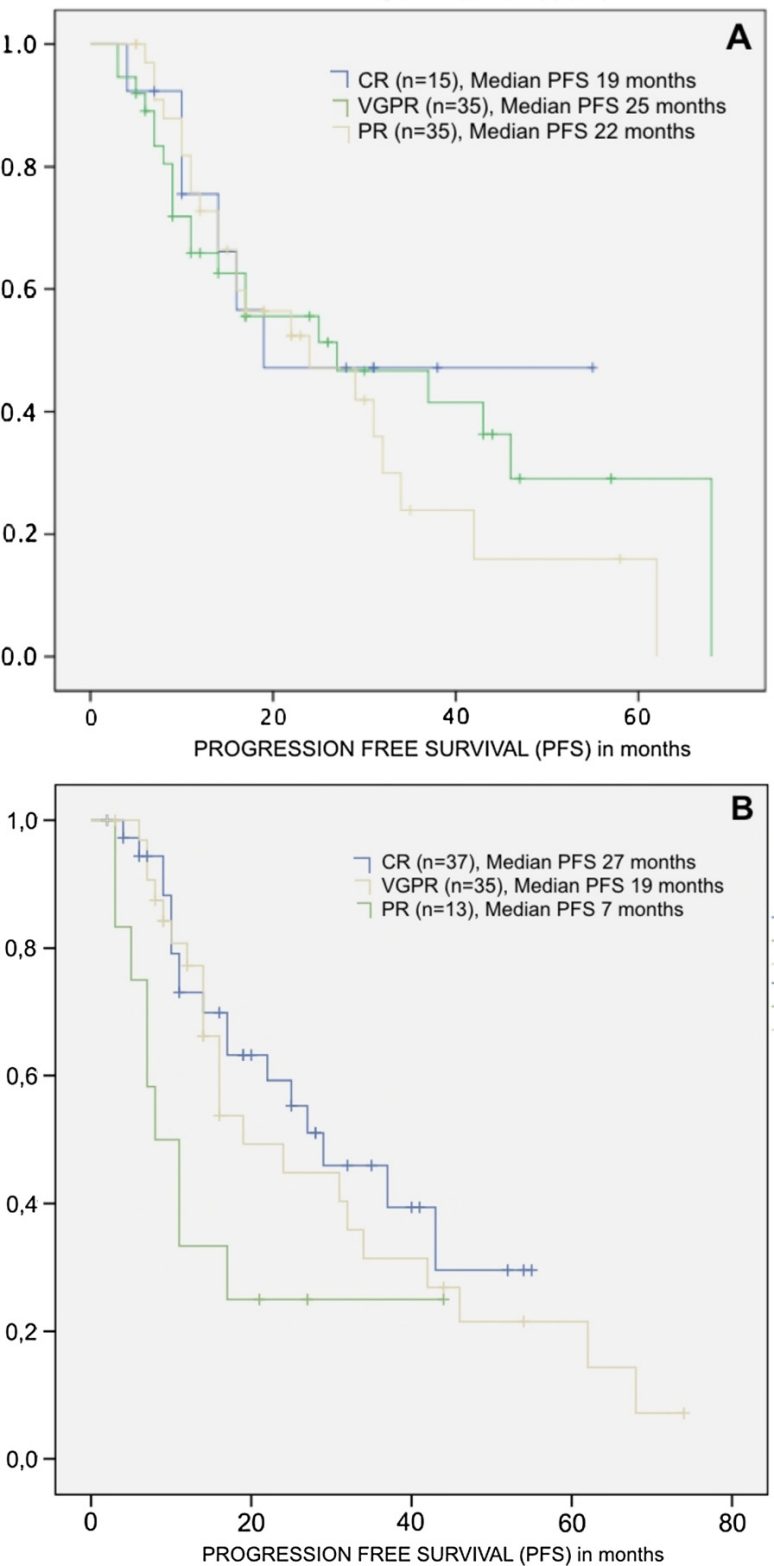
**Kaplan Meyer curves for Progression free survival from patients with Multiple Myeloma according to the response achieved after induction chemotherapy (A) – CR vs VGPR, p = 0.485; CR vs RP, p = 0.710; VGPR vs PR, p = 0.508) and after autologous stem cell transplant (B) - CR vs VGPR, p = 0.529; CR vs RP p = 0.034; VGPR vs RP, p = 0.049.** (*CR – complete response; VGPR – very good partial response; PR – partial response).*

No statistically significant difference was found in PFS of patients in CR at the end of induction as compared to patients in VGPR (27 *vs* 19 months, p = 0.485) (Figure 2A). After ASCT, patients with CR have higher PFS compared to patients with PR (27 *vs*. 7 months, p = 0.034), but not when compared to patients with VGPR (Figure 2B). For OS there is a trend in favour of patients in CR and VGPR as compared to patients in PR, without reaching, however, statistical significance.

**Figure 3:**
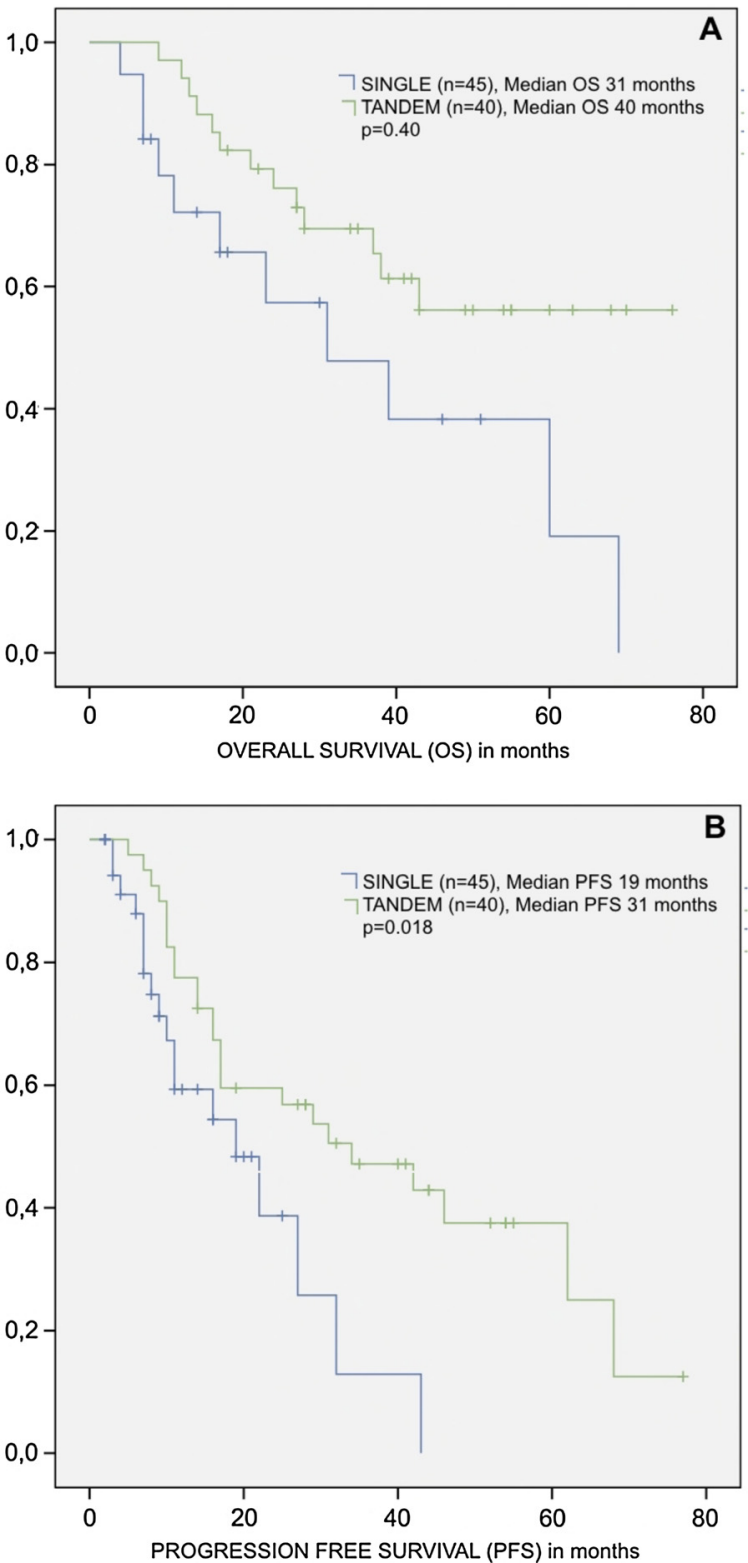
Kaplan Meyer curves for Overall survival (A) and Progression free survival (B) from patients with Multiple Myeloma according to the modality of autologous stem cell transplantation (single or tandem).

The *tandem* modality represented a statistically significant advantage as compared to patients receiving only one transplant. A median PFS of 31 *vs* 19 months was obtained for patients submitted to tandem *vs*. single transplant (p = 0.018), respectively, and a median OS of 40 *vs*. 31 months for *tandem* modality *vs*. single transplant (p = 0.040), respectively (Figure 3).

## Current status

Of the 51 patients who are still attending the transplant follow-up clinic, 25% (n = 21) remain in CR, 1.2% (n = 1) in second CR, 15.5% (n = 13) in VGPR, 2.4% (n = 2) in second VGPR and 16.7% (n = 14) in PR.

## Discussion

Although ASCT is not a curative therapy for MM, it allows an increase in overall survival and progression-free survival, offering to patients a better quality of life for a longer period of time. In our centre, OS and PFS at 5 years were 45.3% and 24.5%, with a maximum follow-up of 9.5 years. These results were not dissimilar from results obtained in other centres [3,9,10,31].

Usually the impact of response at the end of induction chemotherapy in the outcome of transplant is difficult to interpret as the reduced sample size may hamper the establishment of a significant association. However, we had in our series a relatively good rate of CR (17.6%) and VGPR (41.2%), after induction chemotherapy. Regarding the impact of ASCT in response, there was an increase in CR rate (44.7%) as compared to the end of induction, and this reflected in an advantage of 27 *vs* 7 months in PFS as compared to those who achieved only PR, respectively. However, this relationship is not consensual, particularly in regard to OS as results are more controversial amongst several studies [32-36]. One may question the definition of CR in a pathology characterized by a quasi-inevitable relapse [37]; one should probably speak of a good response (CR + VGPR), incomplete response (PR) or no response groups as major determinant conditioning outcome. We did not also take into account the variation in cytogenetic risk and its impact on the achievement of response. Patients with low/medium risk (about 85% of all with MM) seem to have an equal overall survival regardless of obtaining CR, as opposed to high-risk patients, in whom a CR seems to be fundamental [38].

The non-hematologic toxicity related to conditioning is comparable to other series of transplant patients, especially regarding mucositis and infectious complications; TRM was null in our series. The improvement in supportive care and control of infections over the years contributed undoubtedly to this result. In respect to hematological toxicity, there was a successful and consistent engraftment of neutrophils and platelets either in the first or the second ASCT. Forty-four patients received G-CSF in the post-transplant period. However, the neutrophil engraftment kinetics was similar regardless of the use of G-CSF. No advantage in terms of OS and PFS was noted, as mentioned [39].

Forty patients in our series underwent a second programmed transplant of hematopoietic progenitors and this *tandem* modality showed a higher OS and PFS when compared to patients who only underwent a single ASCT. This is a major acknowledge once to date, three randomized trials were conducted attempting to clarify the benefit of this approach [40-42]. The results were inconsistent, mainly due to methodological differences that hamper a consensus in this matter. All those studies showed an advantage in respect to PFS, but only two of them revealed some benefit on OS [41,42]. Completion of a second transplant were feasible in 75% of patients, without higher morbidity and with an unchanging TRM. The IFM94 study showed that the only parameter that could predict the performance of a second transplant would be the answer to the first transplant [43], which was later confirmed by another study by an Italian group [41]. Thus, in view of our results, although those did not derive from a comparative randomized trial, the tandem modality might be considered to be of benefit for most patients.

New drugs, firstly introduced in the treatment of relapse, were quickly incorporated into induction protocols. Several studies aimed at reaching better response rates after induction with these new drugs [44,45]. In our study, we did not find any significant increase in PFS and OS in patients who underwent ASCT after bortezomib-containing induction protocol as compared to other induction protocols. However this comparison is merely historical and no randomization was done between the induction regimens.

Other therapeutic option that is being assessed for MM patients by some groups is the auto-allogeneic tandem transplantation, but the results are controversial. It may overcome negative prognostic effect cytogenetic high risk MM with a longer survival rates as suggested by some groups [46,47], but in others the high incidence of graft-versus-host-disease as well as the high TRM is a major limitation [48,49]. In fact our data supports the feasibility of a tandem auto-auto with low morbidity and mortality.

Despite the results presented, and the well consolidated importance of ASCT in MM, the upfront role of autologous stem cell transplant on MM treatment has been sometimes questioned, under the argument that combination of 3 or 4 drugs might achieve CR in many cases, reserving transplant to salvage therapy after relapse or disease progression [50-52], but this is far from being proved. Currently two randomized trials are being conducted by the *European Myeloma Network* groups and the *FrancoAmerican* consortium *MFI* / *DFCI 2009*, to assess that question [53]. However, while these studies do not show results, ASCT after induction chemotherapy with double or triple combination of new drugs remains the *gold standard* treatment for MM.

## Competing interests

The authors reported no potential competing interests.

## Authors’ contributions

RB was the principal investigator and takes primary responsibility for the paper. RB collected the data, wrote the article and performed statistical analysis. FT collected the data and coordinated the research. RB, FT and JEG interpreted data. All authors have read and approved the final manuscript.
